# Preoperative Education for Less Outpatient Pain after Surgery (PELOPS trial) in orthopedic patients—study protocol for a randomized controlled trial

**DOI:** 10.1186/s13063-022-06387-6

**Published:** 2022-05-21

**Authors:** Mikhail Dziadzko, Axelle Bouteleux, Raphael Minjard, Jack Harich, Fanny Joubert, Pierre Pradat, Solene Pantel, Frederic Aubrun

**Affiliations:** 1 Department of Anesthesiology and Intensive Care Medicine, Croix Rousse University Hospital, Hospices Civils de Lyon, Lyon, France; 2 RESHAPE Lab, U1920, INSERM and Claude Bernard Lyon 1 University, Lyon, France; 3 Department of Pain treatment, Croix Rousse University Hospital, Hospices Civils de Lyon, Lyon, France; 4 Center of Research in Clinical Psychopathology and Psychology (CRPPC) University Lumière Lyon 2, Lyon, France; 5 BS Systems Engineering, Georgia Institute of Technology, Atlanta, GA USA; 6 Center for Clinical Research, Croix Rousse University Hospital, Hospices Civils de Lyon, Lyon, France

**Keywords:** Empowerment, Outpatient, Orthopedic surgery, Patient education, Postoperative pain, Pain relief

## Abstract

**Background:**

Successful pain management after outpatient surgery requires proper education leading to correct decisions on the analgesics use at home. Despite different strategies adopted, up to ½ of patients receive little or no information about the treatment of postoperative pain, 1/3 of them are not able to follow postoperative analgesia instructions. This leads to higher rates of unmet needs in pain treatment, post-discharge emergency calls, and readmissions. Structured educational interventions using psychological empowering techniques may improve postoperative pain management. We hypothesize that preoperative education on use of an improved pain scale to make correct pain management decisions will improve the quality of post-operative pain management at home and reduce analgesics-related side effects.

**Methods:**

A total of 414 patients scheduled for an outpatient orthopedic surgery (knee/shoulder arthroscopic interventions) are included in this randomized (1:1) controlled trial. Patients in the control arm receive standard information on post-discharge pain management. Patients in the experimental arm receive structured educational intervention based on the rational perception of postoperative pain and discomfort (anchoring and improved pain scale), and the proper use of analgesics. There is no difference in post-discharge analgesics regimen in both arms. Patients are followed for 30 days post-discharge, with the primary outcome expressed as total pain relief score at 5 days. Secondary outcomes include the incidence of severe pain during 30 days, changes in sleep quality (Pittsburg Sleep Quality Assessment), and patients’ perception of postoperative pain management assessed with the International Pain Outcomes questionnaire at day 30 post-discharge.

**Discussion:**

The developed intervention, based on an improved pain scale, offers the advantages of being non-surgery-specific, is easily administered in a short amount of time, and can be delivered individually or in-group, by physicians or nurses.

**Trial registration:**

ClinicalTrials.govNCT03754699. Registered on November 27, 2018.

**Supplementary Information:**

The online version contains supplementary material available at 10.1186/s13063-022-06387-6.

## Administrative information

Note: the numbers in curly brackets in this protocol refer to SPIRIT checklist item numbers. The order of the items has been modified to group similar items (see http://www.equator-network.org/reporting-guidelines/spirit-2013-statement-defining-standard-protocol-items-for-clinical-trials/).

**Table:** 

Title {1}	Preoperative Education for Less Outpatient Pain after Surgery (PELOPS trial) in orthopedic patients– study protocol for a randomized controlled trial
Trial registration {2a and 2b}.	NCT03754699 at ClinicalTrials.gov https://clinicaltrials.gov/ct2/show/NCT03754699 November 27, 2018
Protocol version {3}	Version 4 from 9/12/2020
Funding {4}	Fondation Apicil, France and the French Society of Anesthesiologistes (SFAR – Societé Francaise d’Anesthesie et Reanimation) – PrixSFAR/APICIL-2017
Author details {5a}	a: Department of Anesthesiology and Intensive Care Medicine, Croix Rousse University Hospital, Hospices Civils de Lyon, Lyon, France b: RESHAPE Lab, U1920, INSERM and Claude Bernard Lyon 1 University, Lyon, France c: Department of Pain treatment, Croix Rousse University Hospital, Hospices Civils de Lyon, Lyon, France d: Center of Research in Clinical Psychopathology and Psychology (CRPPC) University Lumière Lyon 2, Lyon, France e: BS Systems Engineering, Georgia Institute of Technology, Atlanta, GA, USA f: Center for Clinical Research, Croix Rousse University Hospital, Hospices Civils de Lyon, Lyon, France
Name and contact information for the trial sponsor {5b}	Fondation APICIL 21, place Bellecour 69002 Lyon
Role of sponsor {5c}	Funder has no role in study design; collection, management, analysis, and interpretation of data; writing of the report; and the decision to submit the report for publication

## Introduction

### Background and rationale {6a}

The rate of ambulatory surgery varies between 30% and more than half of all surgery in high-income countries involving all age patients [1, 2]. Postoperative pain management and nausea-vomiting are the main barriers for the successful outpatient track and are the main issues for the first 72 h after discharge [3, 4].

Multiple approaches for the outpatient pain management include preemptive and multimodal anesthesia, pharmacological treatment, coordination and follow-up solutions, ambulatory pain specialist involvement, and patient education [5–7]. Caregivers should provide proper information covering the modalities of post-discharge analgesia [8].

Structured education intervention using psychological techniques to enhance engagement and behavior was found to be effective in different chronic conditions [9, 10]. For instance, pain knowledge and attitude improvements have a positive effect on pain intensity in cancer patients who are already on opioid therapy at home [11]. In hospitalized orthopedic patients such intervention improves patient-reported outcomes (PRO) [12]. However, no robust evidence is available on its real benefit in the outpatient settings [13].

Self-management of postoperative pain at home is based on auto-evaluation of pain intensity. Pain intensity scales for auto-evaluation (11 items numeric, analogs or visual, simplified visual pain scales) are proposed to patients in order to help manage pain control at home. However, at least 40% of patients are unable to use it correctly in extra hospital settings [14], and this inability is more marked in a vulnerable population [15, 16].

Preoperative instruction on the use of a pain scale was found to improve the patient's ability to self-report pain [17]. Nevertheless, patients in an outpatient clinic would prefer other than a simple numeric scale to assess their pain [18]. Patients choose to use comprehensive pain scales, which rationalize their perception of pain and discomfort, probably preventing from emotional amplification and catastrophizing [19].

### Objectives {7}

We hypothesize that an educational intervention based on the rational perception of postoperative pain and discomfort, and the proper use of analgesics would improve the quality of pain management at home and reduce analgesics-related side effects.

The study evaluates the effectiveness of an educational intervention on pain relief at home in outpatient orthopedic surgery, compared to standard care. The pain relief is assessed during the first five post-discharge days (primary outcome), then on the 10th, 20th and 30th days after surgery (secondary outcome). The prevalence of severe pain (as reported by participants), medication use, side effect of medication, changes in sleep quality, the incidence of neuropathic pain at day 30 after surgery, and overall satisfaction at day 30 will be assessed as a part of secondary objectives.

### Trial design {8}

PELOPS is a single-center prospective randomized controlled two-group superiority trial with parallel assignment. This manuscript describes the study protocol and the beginning of the trial in the setting of outpatient orthopedic surgery

## Methods: Participants, interventions, and outcomes

### Study setting {9}

This study is performed at the Hopital de la Croix Rousse, Hospices Civils de Lyon, tertiary university hospital in France. Three departments are involved in the trial - Department of Orthopedic Surgery, Department of Anesthesia, and Outpatient Surgery Department.

### Eligibility criteria {10}

Adult patients (age more than 18 years old), scheduled for elective outpatient orthopedic surgery in will be recruited. Surgical interventions include knee and shoulder arthroscopy, cruciate ligament repair, and medial patellofemoral ligament (MPFL) repair. These interventions are frequent and are known to produce moderate to severe pain in the early postoperative period [20].

Non-eligible are pregnant or breast-feeding women, patients with contraindication for acetaminophen and anti-inflammatory drugs, with chronic pain beyond the surgical site, outpatient knee and hip arthroplasty (specific education and at-home program already in place), patients without social security coverage, non-French speaking, unable to consent, disagree to participate.

### Who will take informed consent? {26a}

Patients will be recruited during the pre-anesthesia visit by a physician anesthesiologist. After meeting inclusion criteria, each patient will be orally informed about the study protocol first. Agreed patients will sign a written consent form and will be randomized to receive either standard information on postoperative pain management or structured education intervention. A physician anesthesiologist, performing pre-anesthesia evaluation and patients’ inclusion, collects the written consent.

### Additional consent provisions for collection and use of participant data and biological specimens {26b}

In a case of an ancillary study with the data collection not covered in the original informed consent form, the new signed consent has to be obtained from every participant. In a case of retrospective use of already generated data, stored in the institutional Electronic Health Record, all patients should be informed about their data use according to the European General Data Protection Regulation. No biological specimens will be collected in this trial.

## Interventions

### Explanation for the choice of comparators {6b}

#### Standard pain information

The standard recommendation on postoperative pain management for outpatient procedures is based on the recommendation of the French National Agency for Medication and Medical Products Safety (Agence nationale de sécurité du médicament et des produits de santé –ANSM) and the French Society of Anesthesia (SFAR), available through the site of the French national system of health insurance [21, 22].

These recommendations include an example of 11-item numeric pain scale (0 – no pain, 10 – worst imaginable pain) divided into three levels—light to moderate (0 to 4, light orange code), moderate to intense (4 to 7, orange code), and intense to very intense (7 to 10, dark orange code).

The following narration is used:

Your pain treatment has to be taken starting your return home. To evaluate your pain, use the digital scale from 0 to 10. In a case of light to moderate pain (0-4), you should take paracetamol. For moderate to intense pain (4-7), you should take anti-inflammatory or opioid-containing drugs.

The treatment is systematic during first 3 days. Prescribed dosage should be respected.

All patients were free to ask any additional questions about pain management at home.

At the end of visit, a diary for data collection is given to a patient, containing the comparator standard pain scale on the first page, and the narration on the second page.

All parents (both from the control and intervention arms) receive a standardized analgesics prescription for at-home pain management, including acetaminophen, ketoprophen (non-steroid anti-inflammatory drug), weak opioid formulation (based on opium), and proton pump inhibitors (gastric protector).

### Intervention description {11a}

The structured education intervention is derived from the well-known and validated 11-items pain intensity scale. The intervention allows the anchoring of each level of pain scale on the individual patient’s experience. In the case of outpatient surgery, patients are empowered to act on medical (post-surgical pain, functional disability) and social constraints (return home, autonomy, home burden, family, no immediate access to a healthcare provider). Patients receive oral, visual (interaction with a clinician), and material support (diary with scale and script), delivered by a clinician, in a standardized manner.

#### Educational intervention development

A cognitive bias where a particular reference point would influence an individual’s decision is known as “anchoring effect” [23]. In patients who need to evaluate their pain intensity regularly, a pain scale anchors modify final judgment and behavior [24–26], especially in chronic pain [27].

Several “comprehensive” patient-developed pain scales are available. Examples include 11-items based Harich’s Comparative Pain Scale [28] and Mankoski Pain Scale [29]. Although not clinically validated, they are used in different pain-related conditions [30–32]. Both scales use anchors for each level of pain from 1 to 10, as examples of common experience (e.g., “average toothache,” ”pinching the fold of skin”) and the ability of daily functions (e.g., “you can still go to work,” “you can no longer think clearly”).

For the purpose of our trial, we used both scales as a prototype for the Comprehensive Acute Pain Anchoring Scale for the Outpatient Orthopaedic Surgery.

We first translated both scales into French, using the process of cross-cultural adaptation of self-reported measures [33]. Two translators (informed – with medical English, and non-informed – no medical English knowledge) were solicited. First, French translation was reviewed conjointly by translators to resolve any discrepancies. Furthermore, another two translators with native English, and naïve to the purpose of the trial, produced a backward translation into English, and discrepancies were resolved. The final version was reviewed by all translators and investigators for the accuracy of used terms and tested for the face validity [34].

Investigators (MD, AB, and RM) used the final translation to create a simplified pain anchor-based scale on the ability to have regular daily activities (home care, meals, sleep). The French version of the produced scale is presented in Additional file 1: Appendix 1.

Based on this scale, an educational intervention was developed conjointly with psychologists (MD, RM). The following psychological concepts were used: a recall of the most intense pain experienced in the past (recall and negativity bias, self-experience anchoring) [35], anchoring or calibration of the individual’s pain experience using a new scale (different pain levels anchoring), and positive suggestions targeting pain management at home [36].

A standard narration was developed containing nine steps (Additional file 1: Appendix 2):

1. Enounce of the purpose
2. Recall of the past pain experience
3. Quantification of the experience on the scale from 0 (no pain) to 10 (worst imaginable)
4. Qualification of the past pain experience (what was pain felt like)
5. Request to discriminate the weak, moderate, and strong pain
6. Anchoring of the recalled pain experience with the experimental scale
7. Principles of the transition of mild to moderate and moderate to severe pain explained
8. Pain management principle explained (pain relief, anticipation, medication)
9. Reinsurance

Therefore, the educative intervention consists in standard narration and presentation of the anchoring scale. It is comprehensive in scope, standardized, flexible in content and responsive to an individual’s clinical and psychological needs, and adaptable to the patient’s educational and cultural background [37].

#### Educational intervention delivery

Educational intervention consists of the interaction based on standardized narration and demonstration of the anchoring scale. A standardized narration and sequence is used (Additional file 1: Appendix 2).

Again, all patients were free to ask any additional questions about pain management at home. The average time for this intervention is from 5 to 10 min. At the end of visit, a diary for data collection is given to a patient, containing the experimental scale on the first page, and the narration on the second page.

### Criteria for discontinuing or modifying allocated interventions {11b}

Patients who may wish not to continue the study (consent withdrawal) are free to terminate their participation at any moment. The principal investigator may suspend or withdraw a patient participation for any reason that may conflict with the interest of the patient.

### Strategies to improve adherence to interventions {11c}

Instructions about the purpose of the study and the importance to fill in and to return the diary are thoroughly provided during the inclusion visit. Participating patients will receive a text message via their private e-mail or phone (short message service, SMS) on the day, where their action to fill in the diary is necessary (day 0–day 5, days 10, 20, and 30). The last message on day 30 contains instructions on how to return their diary. Patients are expected to bring the filled diary on day 45 (scheduled surgical visit) or send it by post. Patients are provided with prepaid envelopes in order to send the diary if no postsurgical visit is scheduled on day 45.

### Relevant concomitant care permitted or prohibited during the trial {11d}

Participants are provided with a standard pain treatment order including acetaminophen, ketoprofen, and weak opioids. They are allowed to use any additional medication or non-pharmacological pain relief, in such cases, this should be traced in the diary. In France, the access to non-steroid and opioid analgesics is highly regulated, so the chance that enrolled patients will considerably modify their home treatment is weak. Hypothetically, patients hospitalized during the follow-up period, or deceased, will be considered as lost for follow-up and therefore excluded.

### Provisions for post-trial care {30}

Enrolled patients are covered by indemnity for non-negligent harm through the standard procedure. Hospices Civils de Lyon have insurance to cover for non-negligent harm associated with the protocol, including additional health care, compensation, or damages.

### Outcomes {12}

The primary outcome is the pain relief measured with a percentage of maximum total pain relief (TOTPAR) over 5 days. TOTPAR is a time-weighted measure of total area under the pain relief curve. This measurement assimilates iterative assessments of a subject’s pain over the duration of the study.

The secondary outcome includes:

1. TOTPAR at day 10, day 20, and day 30 after surgery
2. The incidence of severe pain (defined as “severe pain” by the patient)
3. Type, quantity, and doses of taken analgesics
4. Neuropathic pain prevalence at day 30, detected with DN4 scale [38]
5. The sleep quality measured with Pittsburgh Sleep Quality Index (PSQI) [39] and compared at inclusion day and at the 30th postoperative day
6. Patients’ perception of postoperative pain management evaluated at with the International Pain Outcome (IPO) questionnaire [40] on day 30 after surgery

### Participant timeline {13}

Patients are followed for 30 days after the discharge from the hospital (Table 1).

**Table 1:** Timeline for patients included in the trial

Actions	Days of study										
	–30 to 0	Surgery	1	2	3	4	5	10	20	30	45
Inclusion/exclusion criteria check	X										
Information and written consent signed	X										
Randomization	X										
Neuropathic pain evaluation	X									X	
Sleep Quality Evaluation	X									X	
International Pain Outcome Questionnaire										X	
At-home diary : - Pain relief, % - Medication taken - Side effects		X	X	X	X	X	X	X	X	X	
Recall SMS/email			X				X	X	X	X	
Diary collection											X

*SMS* short message service

### Sample size {14}

In the beginning of our study, we were not able to identify any randomized study of educational intervention with TOTPAR as a primary endpoint. It has been reported, that more than 30% of orthopedic patients suffer from “important” pain (e.g., >6/10) during the first 72 h post-discharge [41, 42]. A 2-point change in the 11-item scale or 30% of the difference in pain intensity measurement is defined as clinically significant [43].

We analyzed the literature, reporting the effect of preoperative education on postoperative orthopedic pain in outpatient settings, and available on the year of the study development. Two studies reported the clinically significant effect from the preoperative education, with calculated OR 1.77 [95%CI 1.12–2.79] to 1.97 [95%CI 1.09–3.57] [44, 45]. However, the quality analysis of these studies demonstrated important heterogeneity, the absence of power calculation, small clinical effects, and design flaws.

As we were not able to find any relevant data for the sample size calculation, we have arbitrarily chosen the OR=2 from the effect of preoperative education, which will produce the decrease of proportion of patients with “important” pain from 30 to 17.6%. Giving this assumption, with the risk alpha of 5% and the power at 80%, allocation 1:1, the calculated sample size was 368 patients. We estimate the loss to follow-up at 10%, therefore the 410 patients will be finally enrolled.

### Recruitment {15}

Our institution performs at least 50 orthopedic outpatient procedures monthly. Anesthesiologists’ team (6 practitioners) systematically evaluates all patients at least 48 h before the planned surgery. As we do not expect to recruit every patient, we would need at least 48 months for the inclusion target accrual.

### Assignment of interventions: allocation

#### Sequence generation {16a}

Enrolled patients will be randomly assigned to a control or interventional group with a 1:1 allocation using Ennov Clinical® software (Paris, France) with an embedded IWRS (Interactive Web Response System) randomization system. Patients will be stratified by the type of surgery (knee, hip, or shoulder isolated arthroscopy, MPFL, Kenneth Jones or Hamstring anterior cruciate ligaments repair, posterior cruciate ligaments repair, Latarjet open shoulder procedure, and video-assisted rotator cuff repair) and type of anesthesia (spinal, general, combined general and regional anesthesia). The configuration of the randomization and the implementation of the randomization list in the software were made by the statistician who is not involved in the process of patient inclusion and randomization. The list was created with variable block size using nQuery Advisor®, 7.0 (Statistical Solutions Ltd, Cork, Ireland). No one, except the statistician, has access to the randomization list.

#### Concealment mechanism {16b}

The result of randomization will be available after obtaining the patient’s consent, and before study group assignment, using an online, central randomization service IRWS. Allocation concealment will be ensured, because the IRWS will not issue the group assignment until the patient has been recruited into the trial.

#### Implementation {16c}

Recruiting physician will use IRWS of the Ennov Clinical® software to receive allocation instructions and will give the information about allocation to the patient.

There will be no other changes in anesthesia protocol or postoperative care protocol, the same standard at-home analgesics prescription is provided for all patients at discharge by surgeons.

### Assignment of interventions: blinding

#### Who will be blinded {17a}

Due to the nature of the intervention, the allocation cannot be masked neither for participants nor for physicians or data managers

#### Procedure for unblinding if needed {17b}

Not applicable, no blinding.

### Data collection and management

#### Plans for assessment and collection of outcomes {18a}

A dedicated electronic case report form (e-CRF) within Ennov Clinical® software was created and managed by Clinical Research Department, Hospices Civils de Lyon. The following data are collected upon inclusion: demographics, type of surgery, type of anesthesia, assessment of neuropathic pain with DN4, and assessment of the sleep quality with PSQI. After the discharge, patients fill in the dedicated diary. The prevalence of severe pain (which patients deem to be important) (binary), pain relief (continuous), painkiller administration (binary), and side effects from painkiller (binary) are collected in a time-dependent manner with 30 min grid over 24 h. On day 30, patients fill in the assessment of neuropathic pain with DN4, assessment of the sleep quality with PSQI, and the International Pain Outcome questionnaire (multiple binary, categorical and continuous variables). After the diary completion, patients were asked to send the collected data to the investigators.

#### Plans to promote participant retention and complete follow-up {18b}

After the inclusion and discharge from the hospital, patients receive a short text message and/or e-mail reminders to complete the follow-up on days 0, 1, 2, 3, 4, 5, 10, 20, and 30 post-discharge. A postal stationery envelope (prepayment of postage) with the printed investigator’s return address is given to each patient along with the diary. Patients are invited to return their diary via the post service, or they may bring it to the hospital. Two weeks following the end of study, if there is no diary return, the patient is contacted by the associate research coordinator and invited to send the diary to the investigator.

#### Data management {19}

Upon each diary reception, all data are transcribed to the e-CRF by a dedicated Clinical Research Associates from Clinical Research Department, Hospices Civils de Lyon. The quality of collected data is verified upon monitoring visits. After the study completion, all data will undergo the quality check by the Data Manager. All collected data are confidential and anonymized. Only the principal investigator has access to the de-anonymization tables.

#### Confidentiality {27}

All study-related information is stored in the secured institutional Electronic Health Record system and electronic Clinical Research Form system *Ennov*, controlled by our institution. The access to both systems is controlled. All study-related data are pseudo-anonymized, and the deanonymization list is encrypted, stored separately, and available only for investigators.

#### Plans for collection, laboratory evaluation, and storage of biological specimens for genetic or molecular analysis in this trial/future use {33}

Not applicable; no biological sampling nor laboratory investigation is planned for this study.

### Statistical methods

#### Statistical methods for primary and secondary outcomes {20a}

Analyses will be performed on an intention-to-treat basis. All data will be checked for normal distribution, and variables will be presented as mean ± standard deviation (SD) or as the median and interquartile range [IQR] accordingly. Categorical variables will be presented as the number and percentage of the total. To compare the two groups, we will use Fisher’s exact test for qualitative data and the non-parametric Mann-Whitney tests for quantitative variables. A comparison of the two groups at randomization will identify potential bias due to unequal allocation. A multivariate logistic regression analysis will be performed to study conditions independently associated with the response to the intervention and to control potential confounding factors. A 0.05 *p* value will indicate statistical significance. Statistical analyses will be performed using R software (R Foundation for Statistical Computing, Vienna, Austria).

#### Interim analyses {21b}

We do not plan to perform an interim analysis.

#### Methods for additional analyses (e.g., subgroup analyses) {20b}

No additional analyses are planned.

#### Methods in analysis to handle protocol non-adherence and any statistical methods to handle missing data {20c}

No missing data will be accepted for the primary end-point. If there are no measures of pain relief after the postoperative day three registered, but the diary is completed, the TOTPAR will be considered as 100% starting from the next blank page. In a case of missing data for the first three days post-discharge, patients will be replaced by new patients, recruited up to 186-target accrual. Missing data for the secondary endpoints will be examined and the decision guided by a principal investigator and statistician will determine whether these data will be excluded or imputed. In that case, a Multiple Imputation by Chained Equations (MICE) will be performed. A R-software implemented algorithm imputes missing data through an iterative series of predictive models using the other existing variables in the dataset.

#### Plans to give access to the full protocol, participant-level data and statistical code {31c}

The full protocol (French version) is available upon request to the authors. No participant-level data will be shared.

### Oversight and monitoring

#### Composition of the coordinating center and trial steering committee {5d}

This study is promoted and coordinated by the Direction of Clinical Research and Innovation (DRCI) of Hospices Civils de Lyon, France. This structure includes dedicated Clinical Research Associates, who will monitor the study upon EudraLex practice guidelines.

#### Composition of the data monitoring committee, its role, and reporting structure {21a}

The data monitoring committee is a part of the institutional Research Department, and is independent from the sponsor; it has no competing interests regarding the study.

#### Adverse event reporting and harms {22}

The nature of the study (education intervention) is not intended to produce any harm. However, at the last visit, any potential adverse effect will be collected by the responsible surgeon.

#### Frequency and plans for auditing trial conduct {23}

At least 10 monitoring sessions are scheduled—before the first inclusion, every 50 inclusions, and after the last inclusion.

#### Plans for communicating important protocol amendments to relevant parties (e.g., trial participants, ethical committees) {25}

A formal amendment to the protocol will be made for any changes to the protocol that may affect the study (objectives, endpoints, design, participants population, sample sizes, procedures, or significant administrative aspects). These changes will be reported to relevant parties (e.g., trial registry, ethics committee).

#### Dissemination plans {31a}

The results of the trial will be disseminated through international scientific conferences and submitted to highly ranked peer-reviewed journals for publication. The findings will also be shared through regional, national, and international scientific and practical meetings.

## Discussion

PELOPS is a randomized controlled trial comparing a structured educational intervention to a control group that receives standard information on pain self-management at home after discharge from orthopedic outpatient surgery. The primary outcome measurement is focused on the time-dependent variable—TOTPAR score as the most sensible, and not on summarizing pain intensity assessment [46], with a clinically significant threshold [43]. A post-discharge burden incurred by patients at home (side effects of treatment, sleep changes, IPO) is assessed as well, because of its importance [42, 47].

Current evidence does not demonstrate a clear effect of preoperative patient education on outpatient pain control. Paucity and methodology weakness (heterogeneous primary outcomes, unclearly defined population, unclearly described intervention) of published studies preclude the inference [12, 13]. Although not blinded (impossible to mask the intervention), this will be the first to our best knowledge randomized study, using meaningful measurement to evaluate the effectiveness of a standardized educational intervention on pain relief at home. Our intervention targets pain relief, independent and universal outcome, and not focused on a specific type of procedure.

Rising popularity of minimally invasive surgeries, thorough selection of patients, and availability of high-quality short-acting anesthesia has led to a dramatic decrease in hospital stay for numerous procedures. The low-risk complications and benefits associated with reduced cost and less hospital stay have driven the growth of the market. Arthroplasty, spine fusion, colorectal resection, and open prostatectomy—procedures associated with important potential for postoperative pain—are more and more performed in ambulatory settings (49). Our study will contribute to the growing evidence base for non-pharmacological pain treatment optimization, as we do not modify surgical analgesics prescriptions. If the study hypothesis is supported, the findings will provide the evidence and the base for educational intervention development in other than orthopedics specialties. As our intervention is not specialist-based, a wide range of clinicians can provide it, individually or in groups, before surgery.

## Trial status

At the date of the manuscript revision, the protocol version 4 from 29/12/2020 was effective. A total of 256 patients were recruited.

## Supplementary Information

**Additional file 1: Appendix 1.** Comprehensive Anchoring Scale for Outpatient Orthopedic Surgery. **Appendix 2.** Scandalized script for educational intervention to be used with the scale (Additional file 1: Appendix 1)

**Additional file 2: Appendix 3.** PELOPS Carnet Patients 02

**Additional file 3: Appendix 4.** PELOPS Carnet Patients 01

## Data Availability

The data generated for the final analysis will be stored electronically at the Center of Clinical Research, Hospices Civils de Lyon-France. The full protocol and statistical code will be accessible upon request from the Principal investigator (MD). Participant-level data will not be shared.

## Abbreviations

ANSM: Agence nationale de sécurité du médicament et des produits de santé
DN4: Douleur Neuropathique 4
DRCI: Direction de la Recherche Clinique et Innovations
e-CRF: Electronic case report form
IPO: International Pain Outcome
IQR: Interquartile range
MPFL: Medial patellofemoral ligament
OR: Odds ratio
PELOPS: Preoperative Education for Less Outpatient Pain after Surgery
PRO: Patient-related outcome
PSQI: Pittsburgh Sleep Quality Index
SFAR: Société Française d'Anesthésie et de Réanimation
SMS: Short message service
TOTPAR: Total pain relief
