# Supplementary material for: Preoperative Education for Less Outpatient Pain after Surgery (PELOPS trial) in orthopedic patients—study protocol for a randomized controlled trial
Source: Trials. 2022 May 21;23:422. doi: 10.1186/s13063-022-06387-6 (PMC9123724; doi:10.1186/s13063-022-06387-6)
Supplement: Supplementary file 1 — Additional file 1: Appendix 1. Comprehensive Anchoring Scale for Outpatient Orthopedic Surgery. Appendix 2. Scandalized script for educational intervention to be used with the scale (Additional file 1: Appendix 1) [file 13063_2022_6387_MOESM1_ESM.docx]

Appendix 1 Comprehensive Anchoring Scale for Outpatient Orthopedic Surgery


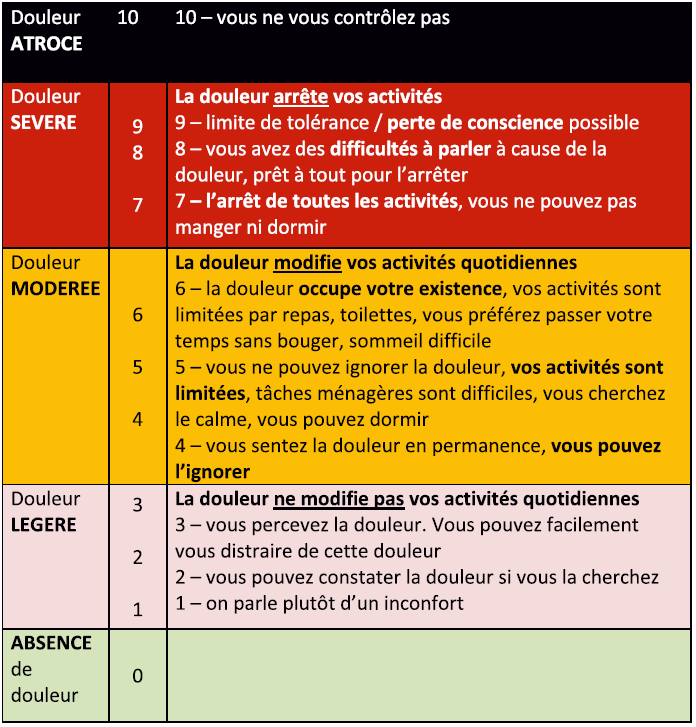


Appendix 2 Scandalized script for educational intervention to be used with the scale (Appendix 1)

|  | Steps | Narration | Action |
| --- | --- | --- | --- |
| 1 | **Enounce/ induction** | To better evaluate and understand your postoperative pain, here we have some questions about you past and present experience. | Narration  /Attention focus |
| 2 | **Recall** | Would you please remember the worst painful experience in the past? | Interaction |
| 3 | **Personal quantification** | Would you please give a score from zero (no paint) to ten (worst imaginable pain) for that pain you had in the past? | Interaction |
| 4 | **Personal qualification** | Could you please describe that pain? How it felt like? | Interaction |
| 5 | **Discrimination** | Would you please describe the difference between weak, moderate and strong pain? | Interaction |
| 6 | **Anchoring** | Now we will read the scale. You will give me the most accurate description from the scale that fit your past experience? | Scale demonstration/ Interaction |
| 7 | **Relativization of anchoring** | Our scale has to thresholds. The first is between 3 and 4 – when pain become quite annoying and you cannot ignore it anymore. However, in two cases you can still have some basic activities at home.  Another threshold is between 6 and 7, when no activities possible anymore – no sleep, no meals. | Scale demonstration/ Narration |
| 8 | **Pain management principle** | The objective of your pain management is to have score below 4. For that, you will evaluate your needs and anticipate your activities. You pain may be 2-3 points worse if you will use your operated joint without medication coverage. That is why during first 3 days after surgery you should take you treatment systematically if you are not able to get distracted from your pain. You will have 3 types of drugs available at home – paracetamol, anti-inflammatory, and opioid based pills. You should take the paracetamol and anti-inflammatory drugs systematically. Please privilege opioid drugs if your pain is still annoying, or if you know, that you will use your operated joint actively (physical therapy etc), take it at least 20-30 minutes before the activity. Pay attention not to exceed the maximum recommended dose. | Narration |
| 8 | **Reinsurance** | Your pain will go away with time. Starting from 3^rd^ day after surgery, you may adapt your pain treatment regarding your discomfort and physical activity.  The attitude we propose may help to minimize the pain after surgery, and to diminish possible adverse effects of pain treatment (nausea, vomiting, dizziness, stomach pain)  Do you have any questions? | Narration /Interaction |
