# Supplementary material for: Preoperative Education for Less Outpatient Pain after Surgery (PELOPS trial) in orthopedic patients—study protocol for a randomized controlled trial
Source: Trials. 2022 May 21;23:422. doi: 10.1186/s13063-022-06387-6 (PMC9123724; doi:10.1186/s13063-022-06387-6)
Supplement: Supplementary file 2 — Additional file 2: Appendix 3. PELOPS Carnet Patients 02 [file 13063_2022_6387_MOESM2_ESM.pdf]

Merci pour votre participation!

N’oubliez pas d’apporter ce carnet lors de la visite de suivi chez votre chirurgien

Hospices Civils de Lyon — votre santé, notre engagement

Impact d'une intervention éducative préopératoire
sur la qualité de la gestion de l'analgésie postopératoire
en chirurgie ambulatoire.

PELOPS
(**P**reoperative **E**ducation for **L**ess **O**utpatient **P**ain after **S**urgery)

Promoteur:
Hospices Civils de Lyon
BP 2251
3 quai des Célestins
69229 LYON cedex 02

Investigateur principal:
Dr Mikhaïl DZIADZKO
Service Anesthésie Réanimation
GHN Hôpital de la Croix Rousse
103, grande rue de la Croix-Rousse
Tél : 04 26 10 93 25
e-mail : mikhail.dziadzko@chu-lyon.fr

FONDATION APICIL
reconnue d'utilité publique
Agir contre la douleur

L'étude est soutenue par la Fondation APICIL

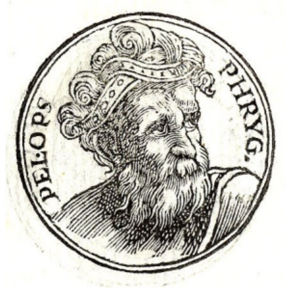

PELOPS

(**P**reoperative **E**ducation for **L**ess **O**utpatient **P**ain after **S**urgery)

Réduire la douleur postoperatoire en ambulatoire

Carnet de suivi

Code Patient : |__||__| / |__||__||__|
(Initiales Nom et Prénom / 3 chiffres selon l’ordre d’inclusion)

Douleur **ATROCE**	10	10 – vous ne vous contrôlez pas
Douleur **SEVERE**	9<br>8<br><br>7	**La douleur arrête vos activités**<br>9 – limite de tolérance / **perte de conscience** possible<br>8 – vous avez des **difficultés à parler** à cause de la douleur, prêt à tout pour l'arrêter<br>**7 – l'arrêt de toutes les activités**, vous ne pouvez pas manger ni dormir
Douleur **MODEREE**	6<br><br>5<br><br>4	**La douleur modifie vos activités quotidiennes**<br>6 – la douleur **occupe votre existence**, vos activités sont limitées par repas, toilettes, vous préférez passer votre temps sans bouger, sommeil difficile<br>5 – vous ne pouvez ignorer la douleur, **vos activités sont limitées**, tâches ménagères sont difficiles, vous cherchez le calme, vous pouvez dormir<br>4 – vous sentez la douleur en permanence, **vous pouvez l'ignorer**
Douleur **LEGERE**	3<br>2<br>1	**La douleur ne modifie pas vos activités quotidiennes**<br>3 – vous percevez la douleur. Vous pouvez facilement vous distraire de cette douleur<br>2 – vous pouvez constater la douleur si vous la cherchez<br>1 – on parle plutôt d'un inconfort
**ABSENCE** de douleur	0	

9. Avez-vous été **autorisé à participer aux décisions concernant le traitement de votre douleur** autant que vous le souhaitiez ?

0	100
||||||||||||||||||||||||||||||||||||||||||||||||||||||||||||||||||||||||||||||||||||||||||||||||||	
Pas du tout	*Complètement*

10. Entourez le chiffre qui décrit le mieux votre **satisfaction du traitement antalgique** depuis votre intervention:

0	100
||||||||||||||||||||||||||||||||||||||||||||||||||||||||||||||||||||||||||||||||||||||||||||||||||	
Extrêmement insatisfait	*Extrêmement satisfait*

11. Avez-vous utilisé ou reçu des **moyens non médicaux pour** soulager votre **douleur**?
☐ Oui ☐ Non

Si oui, **cochez tout** ce qui s'applique:
☐ Application de froid
☐ Méditation
☐ Respiration profonde
☐ Chaleur
☐ Acupuncture
☐ Prière
☐ Parler à l'équipe médicale
☐ Marche
☐ Massage
☐ Parler à des amis ou familiers
☐ Relaxation
☐ Image mentale
☐ Stimulation électrique transcutanée (TENS)
☐ Distraction (comme regarder la TV, écouter de la musique, lire)

☐ Autre (veuillez décrire):

12. Avez-vous eu des **douleurs persistantes pendant 3 mois ou plus** avant de venir à l'hôpital pour cette intervention ? ☐ Oui ☐ Non

a. Si oui sur cette échelle, veuillez indiquer **l'intensité de la douleur** la plupart du temps:

0	100
||||||||||||||||||||||||||||||||||||||||||||||||||||||||||||||||||||||||||||||||||||||||||||||||||	
Aucune	*Pire douleur possible*

b. Si oui, **à quel endroit** était localisée cette **douleur persistante**?

☐ Endroit de l'intervention
☐ Ailleurs
☐ Les deux (endroit de l'intervention et ailleurs)

5. La douleur peut affecter votre humeur et vos émotions.

Sur cette échelle, entourez le chiffre qui décrit le mieux à quel point la **douleur** depuis votre intervention **vous rend:**

a. **Anxieux**

0	100
Pas du tout	*Extrêmement*

b. **Désemparé**

0	100
Pas du tout	*Extrêmement*

c. Avez-vous eu l'un des **effets secondaires** suivants depuis votre intervention?
Cochez "0" si non ; si oui, entourez le chiffre décrivant le mieux la sévérité de cet épisode

- **Nausée**

0	100
Aucun	*Sévère*

- **Somnolence**

0	100
Aucun	*Sévère*

- **Démangeaisons**

0	100
Aucun	*Sévère*

- **Vertige**

0	100
Aucun	*Sévère*

6. Depuis votre intervention, quel **soulagement de votre douleur** avez-vous obtenu ?
Entourez le pourcentage qui décrit le mieux le soulagement de votre douleur par l'ensemble de vos **traitements antalgiques** combinés (traitement médical et non médical)

0	100
Aucun	*Soulagement complet*

7. Auriez-vous aimé avoir reçu **plus d'antalgiques** que vous n'en avez reçu ?
☐ Oui ☐ Non

8. Avez-vous été **informé** sur les possibilités de **traitement de votre douleur**?
☐ Oui ☐ Non

Votre traitement antalgique est à prendre dès le retour à domicile. Pour évaluer votre douleur, vous utilisez l'échelle numérique de 0 à 10.

La grille comprend deux transpositions:
L'une entre **3 et 4** qui correspond au passage entre le moment où vous percevez la douleur mais vous pouvez facilement vous en écarter et le moment où vous ne pouvez plus l'ignorer. Dans les deux cas cette douleur ne modifie pas vos activités quotidiennes.

L'autre transition est entre **6 et 7**. Elle correspond au passage entre le moment où la douleur limite vos activités quotidiennes telles que les repas, les toilettes, le sommeil... et le moment où la douleur empêche toutes activités; vous ne pouvez plus ni manger, ni dormir.

Votre objectif est de maintenir votre douleur postopératoire inférieure à 4. Pour cela vous allez l'évaluer et anticiper les activités que vous avez à faire. Si vous sollicitez l'articulation opérée, ajoutez 2 points à la douleur existante.

Pour cette raison, pendant les 2 premiers jours suivant l'opération vous devez prendre votre traitement de manière systématique lorsque vous n'arrivez pas à vous distraire de la douleur.

Vous avez 2 types de médicaments : les anti-inflammatoires et les médicaments à base de morphine. Il faut prendre les médicaments anti-inflammatoires systématiquement, et privilégier les morphiniques si la douleur ne cède pas ou si vous savez que vous allez solliciter votre articulation (kiné etc) 20-30 min avant.

Attention à ne pas dépasser les doses maximales.

Dès votre retour à domicile, **merci de compléter ce carnet de suivi** le jour de la chirurgie (J0), le lendemain (J1), puis à J2, J3, J4, et J5 puis à J10, J20 et J30 postopératoires.

1. Notez **l'heure d'arrivée** à domicile (simple croix)
2. Faites une croix à chaque fois que vous ressentez une **douleur forte** (ligne « douleur forte »).
3. Notez les différents **niveaux de soulagement ressentis** au cours de la journée (de 0 à 100%).
   Vous devez **<u>au minimum compléter 3 mesures</u>** dans la journée – simple croix
4. Notez les **prises médicamenteuses** réalisées (chiffre=nombre de comprimés)
5. Notez la survenue **d'effets indésirables** liés aux médicaments (1 : Légère ; 2 : Modérée ; 3 : Sévère).

<u>A J30, merci de renseigner</u> :
- Si vous avez consulté votre médecin traitant en raison d'une douleur du site opératoire
- les 3 questionnaires (douleur neuropathique (DN4) ; qualité du sommeil (PSQI) ; le questionnaire International Pain Outcome)

Un rappel vous sera envoyé par SMS à J0, J1, J5, J10, J20.

<u>Votre PLANNING carnet patient</u> :

J0 : |__|__| / |__|__|/ |__|__|__|__| < **(JOUR DE LA CHIRURGIE)**

J1 : |__|__| / |__|__|/ |__|__|__|__| J5 : |__|__| / |__|__|/ |__|__|__|__|

J2 : |__|__| / |__|__|/ |__|__|__|__| J10 : |__|__| / |__|__|/ |__|__|__|__|

J3 : |__|__| / |__|__|/ |__|__|__|__| J20 : |__|__| / |__|__|/ |__|__|__|__|

J4 : |__|__| / |__|__|/ |__|__|__|__| J30 : |__|__| / |__|__|/ |__|__|__|__|

Retour a domicile

DOULEUR FORTE

Je suis soulagé(e)

100%
90%
80%
70%
60%
50%
40%
30%
20%
10%
0%

PARACETAMOL
IZALGY
LAMALINE
CODEINE
TRAMADOL
IBUPROFENE
KETOPROFENE

NAUSEE
VOMISSEMENT
GASTRALGIE
VERTIGES
TROUBLES DE CONCENTRATION
SOMNOLENCE
CONSTIPATION

International Pain Outcome Questionnaire

1. Indiquez la **douleur la plus forte** que vous avez ressentie depuis votre intervention:

0	100
Aucune douleur	*Pire douleur possible*

2. Sur cette échelle, veuillez indiquer la **douleur la plus faible** que vous avez ressentie depuis votre intervention:

0	100
Aucune douleur	*Pire douleur possible*

3. Avec quelle fréquence avez-vous ressenti une **douleur intense** depuis votre intervention ? Cochez votre meilleure estimation du pourcentage de temps pendant lequel vous avez ressenti une douleur intense:

0%	100%
Pas de douleur intense	*Douleur toujours intense*

4. Entourez le chiffre qui décrit le mieux l'intensité avec laquelle la douleur **vous a gêné ou empêché** de faire les activités suivantes depuis votre intervention

a. **mouvements dans votre lit** comme vous tourner, vous asseoir, vous repositionner:

0	100
Pas de gêne	*Empêchement total*

b. **respirer profondément** ou **tousser:**

0	100
Pas de gêne	*Empêchement total*

c. **dormir**:

0	100
Pas de gêne	*Empêchement total*

d. Êtes-vous **sorti du lit** depuis votre intervention? ☐ Oui ☐ Non

Si oui, à quel point la **douleur vous gêne ou vous empêche de faire des activités hors du lit** comme marcher, s'assoir sur une chaise, être debout devant le lavabo:

0	100
Pas de gêne	*Empêchement total*

La qualité de votre sommeil - 02

10. Avez-vous un(e) conjoint(e) ou un(e) camarade de chambre ?

- ☐ Ni l'un, ni l'autre.
- ☐ Oui, dans la même chambre mais pas dans le même lit.
- ☐ Oui, mais dans une chambre différente.
- ☐ Oui, dans le même lit

11. Si vous avez un(e) camarade de chambre ou un(e) conjoint(e), demandez-lui combien de fois le mois dernier vous avez présenté:

	Pas au cours du dernier mois	Moins d'une fois par semaine	Une ou deux fois par semaine	Trois ou quatre fois par semaine
a) Un ronflement fort				
b) De longues pauses respiratoires pendant votre sommeil				
c) Des saccades ou des secousses des jambes pendant que vous dormiez				
d) Des épisodes de désorientation ou de confusion pendant le sommeil				
e) D'autres motifs d'agitation pendant le sommeil				

12	30	13	30	14	30	15	30	16	30	17	30	18	30	19	30	20	30	21	30	22	30	23	30

12	30	13	30	14	30	15	30	16	30	17	30	18	30	19	30	20	30	21	30	22	30	23	30

12	30	13	30	14	30	15	30	16	30	17	30	18	30	19	30	20	30	21	30	22	30	23	30

12	30	13	30	14	30	15	30	16	30	17	30	18	30	19	30	20	30	21	30	22	30	23	30

	0	30	1	30	2	30	3	30	4	30	5	30	6	30	7	30	8	30	9	30
DOULEUR FORTE																				

Je suis soulagé(e)

	0	30	1	30	2	30	3	30	4	30	5	30	6	30	7	30	8	30	9	30
100%																				
90%																				
80%																				
70%																				
60%																				
50%																				
40%																				
30%																				
20%																				
10%																				
0%																				

	0	30	1	30	2	30	3	30	4	30	5	30	6	30	7	30	8	30	9	30
PARACETAMOL																				
IZALGY																				
LAMALINE																				
CODEINE																				
TRAMADOL																				
IBUPROFENE																				
KETOPROFENE																				

	0	30	1	30	2	30	3	30	4	30	5	30	6	30	7	30	8	30	9	30
NAUSEE																				
VOMISSEMENT																				
GASTRALGIE																				
VERTIGES																				
TROUBLES DE CONCENTRATION																				
SOMNOLENCE																				
CONSTIPATION																				

La qualité de votre sommeil - 01

Au cours du mois dernier:

1. Quand êtes-vous habituellement allé vous coucher le soir ? Heure habituelle du coucher: ☐

2. Combien vous a-t-il habituellement fallu de temps (en minutes) pour vous endormir chaque soir ?
Nombre de minutes: ☐

3. Quand vous êtes-vous habituellement levé le matin ? Heure habituelle du lever: ☐

4. Combien d'heures de sommeil effectif avez-vous eu chaque nuit ?
Nombre d'heures de sommeil par nuit: ☐

5. Avec quelle fréquence avez-vous eu des troubles du sommeil car

	Pas au cours du dernier mois	Moins d'une fois/semaine	1-2 fois/ semaine	3 et plus fois par semaine
a) Vous n'avez pas pu vous endormir en moins de 30 mn				
b) Vous vous êtes réveillé au milieu de la nuit ou précocement le matin				
c) Vous avez dû vous lever pour aller aux toilettes				
d) Vous n'avez pas pu respirer correctement				
e) Vous avez toussé ou ronflé bruyamment				
f) Vous avez eu trop froid				
g) Vous avez eu trop chaud				
h) Vous avez eu de mauvais rêves				
i) Vous avez eu des douleurs				
j) Pour d'autre(s) raison(s). Donnez une description:				

6. Comment évalueriez-vous globalement la qualité de votre sommeil ?
☐ Très bonne ☐ Assez bonne ☐ Assez mauvaise ☐ Très mauvaise

7. Combien de fois avez-vous pris des médicaments pour faciliter votre sommeil ?
☐ Pas au cours du dernier mois ☐ Moins d'une fois par semaine
☐ Une ou deux fois par semaine ☐ Trois ou quatre fois par semaine

8. Combien de fois avez-vous eu des difficultés à rester éveillé(e) pendant que vous conduisiez, preniez vos repas, étiez occupé(e) dans une activité sociale ?
☐ Pas au cours du dernier mois ☐ Moins d'une fois par semaine
☐ Une ou deux fois par semaine ☐ Trois ou quatre fois par semaine

9. Avez-vous des problèmes pour vous motiver à faire des choses prévues?
☐ Pas du tout ☐ Seulement un tout petit peu
☐ Certainement ☐ Beaucoup

Depuis votre intervention
avez vous consulté votre medecin traitant? ☐ **OUI** ☐ **NON**

Avez vous toujours des douleurs au niveau de la zone opérée?

☐ **OUI** ☐ **NON** = passez à la page suivante

Si **OUI**

Question 1: La douleur de la zone opérée présente-t-elle une ou plusieurs des caractéristiques suivantes ?	**OUI**	**NON**
1 Brûlure	☐	☐
2 Sensation de froid douloureux	☐	☐
3 Décharges électriques	☐	☐

Question 2: La douleur est-elle associée dans la même région à un ou plusieurs des symptômes suivants ?	**OUI**	**NON**
4 Fourmillements	☐	☐
5 Picotements	☐	☐
6 Engourdissement	☐	☐
7 Démangeaisons	☐	☐

Question 3: La douleur est-elle localisée dans un territoire où l'examen met en évidence ?	**OUI**	**NON**
8 Une diminution de la sensibilité au toucher	☐	☐
9 Une diminution de la sensibilité à la piqûre	☐	☐

Question 4: La douleur est-elle provoquée ou augmentée par	**OUI**	**NON**
10 Le frottement	☐	☐

10	30	11	30	12	30	13	30	14	30	15	30	16	30	17	30	18	30	19	30	20	30	21	30	22	30	23	30

10	30	11	30	12	30	13	30	14	30	15	30	16	30	17	30	18	30	19	30	20	30	21	30	22	30	23	30

10	30	11	30	12	30	13	30	14	30	15	30	16	30	17	30	18	30	19	30	20	30	21	30	22	30	23	30

10	30	11	30	12	30	13	30	14	30	15	30	16	30	17	30	18	30	19	30	20	30	21	30	22	30	23	30

	0	30	1	30	2	30	3	30	4	30	5	30	6	30	7	30	8	30	9	30	10	30	11	30	12	30	13	30	14	30	15	30	16	30	17	30	18	30	19	30	20	30	21	30	22	30	23	30
DOULEUR FORTE																																																

Je suis soulagé(e)

	0	30	1	30	2	30	3	30	4	30	5	30	6	30	7	30	8	30	9	30	10	30	11	30	12	30	13	30	14	30	15	30	16	30	17	30	18	30	19	30	20	30	21	30	22	30	23	30
100%																																																
90%																																																
80%																																																
70%																																																
60%																																																
50%																																																
40%																																																
30%																																																
20%																																																
10%																																																
0%																																																

	0	30	1	30	2	30	3	30	4	30	5	30	6	30	7	30	8	30	9	30	10	30	11	30	12	30	13	30	14	30	15	30	16	30	17	30	18	30	19	30	20	30	21	30	22	30	23	30
PARACETAMOL																																																
IZALGY																																																
LAMALINE																																																
CODEINE																																																
TRAMADOL																																																
IBUPROFENE																																																
KETOPROFENE																																																

	0	30	1	30	2	30	3	30	4	30	5	30	6	30	7	30	8	30	9	30	10	30	11	30	12	30	13	30	14	30	15	30	16	30	17	30	18	30	19	30	20	30	21	30	22	30	23	30
NAUSEE																																																
VOMISSEMENT																																																
GASTRALGIE																																																
VERTIGES																																																
TROUBLES DE CONCENTRATION																																																
SOMNOLENCE																																																
CONSTIPATION																																																

	0	30	1	30	2	30	3	30	4	30	5	30	6	30	7	30	8	30	9	30	10	30	11	30	12	30	13	30	14	30	15	30	16	30	17	30	18	30	19	30	20	30	21	30	22	30	23	30
DOULEUR FORTE																																																

Je suis soulagé(e)

	0	30	1	30	2	30	3	30	4	30	5	30	6	30	7	30	8	30	9	30	10	30	11	30	12	30	13	30	14	30	15	30	16	30	17	30	18	30	19	30	20	30	21	30	22	30	23	30
100%																																																
90%																																																
80%																																																
70%																																																
60%																																																
50%																																																
40%																																																
30%																																																
20%																																																
10%																																																
0%																																																

	0	30	1	30	2	30	3	30	4	30	5	30	6	30	7	30	8	30	9	30	10	30	11	30	12	30	13	30	14	30	15	30	16	30	17	30	18	30	19	30	20	30	21	30	22	30	23	30
PARACETAMOL																																																
IZALGY																																																
LAMALINE																																																
CODEINE																																																
TRAMADOL																																																
IBUPROFENE																																																
KETOPROFENE																																																

	0	30	1	30	2	30	3	30	4	30	5	30	6	30	7	30	8	30	9	30	10	30	11	30	12	30	13	30	14	30	15	30	16	30	17	30	18	30	19	30	20	30	21	30	22	30	23	30
NAUSEE																																																
VOMISSEMENT																																																
GASTRALGIE																																																
VERTIGES																																																
TROUBLES DE CONCENTRATION																																																
SOMNOLENCE																																																
CONSTIPATION																																																

	0	30	1	30	2	30	3	30	4	30	5	30	6	30	7	30	8	30	9	30	10	30	11	30	12	30	13	30	14	30	15	30	16	30	17	30	18	30	19	30	20	30	21	30	22	30	23	30
DOULEUR FORTE																																																

Je suis soulagé(e)	0	30	1	30	2	30	3	30	4	30	5	30	6	30	7	30	8	30	9	30	10	30	11	30	12	30	13	30	14	30	15	30	16	30	17	30	18	30	19	30	20	30	21	30	22	30	23	30
100%																																																
90%																																																
80%																																																
70%																																																
60%																																																
50%																																																
40%																																																
30%																																																
20%																																																
10%																																																
0%																																																

	0	30	1	30	2	30	3	30	4	30	5	30	6	30	7	30	8	30	9	30	10	30	11	30	12	30	13	30	14	30	15	30	16	30	17	30	18	30	19	30	20	30	21	30	22	30	23	30
PARACETAMOL																																																
IZALGY																																																
LAMALINE																																																
CODEINE																																																
TRAMADOL																																																
IBUPROFENE																																																
KETOPROFENE																																																

	0	30	1	30	2	30	3	30	4	30	5	30	6	30	7	30	8	30	9	30	10	30	11	30	12	30	13	30	14	30	15	30	16	30	17	30	18	30	19	30	20	30	21	30	22	30	23	30
NAUSEE																																																
VOMISSEMENT																																																
GASTRALGIE																																																
VERTIGES																																																
TROUBLES DE CONCENTRATION																																																
SOMNOLENCE																																																
CONSTIPATION																																																

	0	30	1	30	2	30	3	30	4	30	5	30	6	30	7	30	8	30	9	30	10	30	11	30	12	30	13	30	14	30	15	30	16	30	17	30	18	30	19	30	20	30	21	30	22	30	23	30
DOULEUR FORTE																																																

Je suis soulagé(e)

	0	30	1	30	2	30	3	30	4	30	5	30	6	30	7	30	8	30	9	30	10	30	11	30	12	30	13	30	14	30	15	30	16	30	17	30	18	30	19	30	20	30	21	30	22	30	23	30
100%																																																
90%																																																
80%																																																
70%																																																
60%																																																
50%																																																
40%																																																
30%																																																
20%																																																
10%																																																
0%																																																

	0	30	1	30	2	30	3	30	4	30	5	30	6	30	7	30	8	30	9	30	10	30	11	30	12	30	13	30	14	30	15	30	16	30	17	30	18	30	19	30	20	30	21	30	22	30	23	30
PARACETAMOL																																																
IZALGY																																																
LAMALINE																																																
CODEINE																																																
TRAMADOL																																																
IBUPROFENE																																																
KETOPROFENE																																																

	0	30	1	30	2	30	3	30	4	30	5	30	6	30	7	30	8	30	9	30	10	30	11	30	12	30	13	30	14	30	15	30	16	30	17	30	18	30	19	30	20	30	21	30	22	30	23	30
NAUSEE																																																
VOMISSEMENT																																																
GASTRALGIE																																																
VERTIGES																																																
TROUBLES DE CONCENTRATION																																																
SOMNOLENCE																																																
CONSTIPATION																																																

	0	30	1	30	2	30	3	30	4	30	5	30	6	30	7	30	8	30	9	30	10	30	11	30	12	30	13	30	14	30	15	30	16	30	17	30	18	30	19	30	20	30	21	30	22	30	23	30
DOULEUR FORTE																																																

Je suis soulagé(e)

	0	30	1	30	2	30	3	30	4	30	5	30	6	30	7	30	8	30	9	30	10	30	11	30	12	30	13	30	14	30	15	30	16	30	17	30	18	30	19	30	20	30	21	30	22	30	23	30
100%																																																
90%																																																
80%																																																
70%																																																
60%																																																
50%																																																
40%																																																
30%																																																
20%																																																
10%																																																
0%																																																

	0	30	1	30	2	30	3	30	4	30	5	30	6	30	7	30	8	30	9	30	10	30	11	30	12	30	13	30	14	30	15	30	16	30	17	30	18	30	19	30	20	30	21	30	22	30	23	30
PARACETAMOL																																																
IZALGY																																																
LAMALINE																																																
CODEINE																																																
TRAMADOL																																																
IBUPROFENE																																																
KETOPROFENE																																																

	0	30	1	30	2	30	3	30	4	30	5	30	6	30	7	30	8	30	9	30	10	30	11	30	12	30	13	30	14	30	15	30	16	30	17	30	18	30	19	30	20	30	21	30	22	30	23	30
NAUSEE																																																
VOMISSEMENT																																																
GASTRALGIE																																																
VERTIGES																																																
TROUBLES DE CONCENTRATION																																																
SOMNOLENCE																																																
CONSTIPATION																																																

	0	30	1	30	2	30	3	30	4	30	5	30	6	30	7	30	8	30	9	30	10	30	11	30	12	30	13	30	14	30	15	30	16	30	17	30	18	30	19	30	20	30	21	30	22	30	23	30
DOULEUR FORTE																																																

Je suis soulagé(e)

	0	30	1	30	2	30	3	30	4	30	5	30	6	30	7	30	8	30	9	30	10	30	11	30	12	30	13	30	14	30	15	30	16	30	17	30	18	30	19	30	20	30	21	30	22	30	23	30
100%																																																
90%																																																
80%																																																
70%																																																
60%																																																
50%																																																
40%																																																
30%																																																
20%																																																
10%																																																
0%																																																

	0	30	1	30	2	30	3	30	4	30	5	30	6	30	7	30	8	30	9	30	10	30	11	30	12	30	13	30	14	30	15	30	16	30	17	30	18	30	19	30	20	30	21	30	22	30	23	30
PARACETAMOL																																																
IZALGY																																																
LAMALINE																																																
CODEINE																																																
TRAMADOL																																																
IBUPROFENE																																																
KETOPROFENE																																																

	0	30	1	30	2	30	3	30	4	30	5	30	6	30	7	30	8	30	9	30	10	30	11	30	12	30	13	30	14	30	15	30	16	30	17	30	18	30	19	30	20	30	21	30	22	30	23	30
NAUSEE																																																
VOMISSEMENT																																																
GASTRALGIE																																																
VERTIGES																																																
TROUBLES DE CONCENTRATION																																																
SOMNOLENCE																																																
CONSTIPATION																																																

5ième jour postoperatoire - J5

	0	30	1	30	2	30	3	30	4	30	5	30	6	30	7	30	8	30	9	30	10	30	11	30	12	30	13	30	14	30	15	30	16	30	17	30	18	30	19	30	20	30	21	30	22	30	23	30
DOULEUR FORTE																																																

Je suis soulagé(e)

	0	30	1	30	2	30	3	30	4	30	5	30	6	30	7	30	8	30	9	30	10	30	11	30	12	30	13	30	14	30	15	30	16	30	17	30	18	30	19	30	20	30	21	30	22	30	23	30
100%																																																
90%																																																
80%																																																
70%																																																
60%																																																
50%																																																
40%																																																
30%																																																
20%																																																
10%																																																
0%																																																

	0	30	1	30	2	30	3	30	4	30	5	30	6	30	7	30	8	30	9	30	10	30	11	30	12	30	13	30	14	30	15	30	16	30	17	30	18	30	19	30	20	30	21	30	22	30	23	30
PARACETAMOL																																																
IZALGY																																																
LAMALINE																																																
CODEINE																																																
TRAMADOL																																																
IBUPROFENE																																																
KETOPROFENE																																																

	0	30	1	30	2	30	3	30	4	30	5	30	6	30	7	30	8	30	9	30	10	30	11	30	12	30	13	30	14	30	15	30	16	30	17	30	18	30	19	30	20	30	21	30	22	30	23	30
NAUSEE																																																
VOMISSEMENT																																																
GASTRALGIE																																																
VERTIGES																																																
TROUBLES DE CONCENTRATION																																																
SOMNOLENCE																																																
CONSTIPATION																																																
